# Cardiac MRI utilizing late gadolinium enhancement (LGE) and T1 mapping in the detection of radiation induced heart disease

**DOI:** 10.1186/s40959-020-00061-z

**Published:** 2020-07-01

**Authors:** Anthony Ricco, Alexander Slade, Justin M. Canada, John Grizzard, Franklin Dana, Leila Rezai Gharai, Keith Neiderer, Armando Vera, Antonio Abbate, Elisabeth Weiss

**Affiliations:** 1 Department of Radiation Oncology, Virginia Commonwealth University Health System, 401 College Street, Richmond, VA 23298 USA; 2 Department of Cardiology, Virginia Commonwealth University Health System, Richmond, VA USA; 3 Department of Kinesiology & Health Sciences, Virginia Commonwealth University Health System, Richmond, VA USA; 4 Department of Radiology, Virginia Commonwealth University Health System, Richmond, VA USA

**Keywords:** Cardiotoxicity from radiotherapy, Radiation induced heart disease, Cardiac MRI, Late gadolinium enhancement, T1 mapping

## Abstract

**Background and purpose:**

Radiotherapy has been associated with late dose-dependent cardiovascular toxicity. In this cross-sectional pilot study, radiation dose distributions were correlated with areas of localized and diffuse myocardial fibrosis as measured by novel cardiac MRI (CMR) sequences including late gadolinium enhancement (LGE) and T1 mapping with the goal to identify early markers of myocardial damage.

**Materials and methods:**

Twenty-eight patients with chest tumors including lung, breast, esophagus, and lymphoma underwent CMR per study protocol on average 46.4 months (range 1.7–344.5) after radiotherapy. Patients without pretreatment cardiac history were included if the volume of heart receiving 5 Gy or more was at least 10% (V5Gy ≥ 10%). The association of LGE with cardiac dosimetric factors, clinical factors (e.g., tumor type, smoking history, BMI), and T1 values was analyzed.

**Results:**

Cardiac maximum (Dmax) and mean dose (Dmean) equivalent to doses delivered in 2 Gy fractions (EQD2) were on average 50.9 Gy (range 6.2–108.0) and 8.2 Gy (range 1.0–35.7), respectively, compared to 60.8 Gy (40.8–108.0) and 6.8 Gy (1.8–21.8) among the 9 patients with LGE. Doses were not different between patients with and without LGE (*p* = 0.16 and 0.56, respectively). The average T1 value of the left ventricle myocardium was 1009 ms (range 933–1117). No significant correlation was seen for heart Dmax and Dmean and T1 values (*p* = 0.14 and 0.58, respectively). In addition, no significant association between clinical factors and the development of LGE was identified.

**Conclusions:**

No relation between cardiac doses, the presence of LGE or T1 values was observed. Further study is needed to determine the benefit of CMR for detecting radiotherapy-related myocardial fibrosis.

## Introduction

Cardiac toxicity is a well-established complication of radiotherapy for mediastinal lymphomas [1], lung [2–4], esophageal [5, 6], and left-sided breast tumors [7–9]. The late effects from incidental radiotherapy to the heart include pericarditis, myocarditis, and coronary artery disease [10, 11]. Delivering radiation dose to the heart can increase the risk for a late competing toxicity precipitating non-cancer morbidity and mortality [12]. More recently, cardiac radiation dose has been shown in numerous series to be associated with lower overall survival and early cardiac event rates [13–16].

In spite of decades of research, fundamental questions about the etiology of radiation-induced heart disease (RIHD) remain [17]. Multiple pathophysiologic mechanisms of RIHD exist, including micro- and macrovascular injury, and endothelial cell dysfunction [18, 19]. Microvascular damage decreases capillary density in the myocardium causing ischemia and has the potential to progress towards fibrosis as a reparative response to heart tissue injury [20]. Fibrosis, a common late toxicity of radiotherapy, can eventually cause diastolic dysfunction, pericarditis, arrhythmias, or other clinically apparent RIHD.

Various imaging modalities have been used to evaluate RIHD including echocardiography, stress echocardiography, SPECT perfusion, cardiac computed tomography (cardiac CT), and cardiac magnetic resonance (CMR) imaging [11]. While each modality offers benefits and drawbacks, CMR offers precise visualization of anatomical structures, calculation of cardiac volumes, quantification of systolic/diastolic function, and evaluation of fibrotic changes, all within a noninvasive imaging modality [11].

LGE has become the radiologic non-invasive standard for determining both ischemic and non-ischemic focal fibrosis of the myocardiu [21–23]. Its ability to detect an increased proportion of extracellular space has been clinically useful for imaging chronically infarcted cardiac tissue representing fibrous scar tissue [24]. LGE sequences exploit the relative difference in T1 recovery times between enhancing regions with extracellular contrast accumulation and normal myocardium with more rapid washout. For this reason, more diffuse myocardial fibrosis can go undetected [21, 25]. T1 mapping is a more recently developed CMR protocol aimed at detecting diffuse myocardial fibrosis with numerous other benefits including no necessity for contrast administration, quantitative characterization of fibrosis, and the ability to follow fibrotic changes over time on longitudinal repeat scans [21, 26]. T1 mapping has been previously described as being able to detect myocardial fibrosis with a linear relationship between the quantity of fibrosis and T1 relaxation times [27]. Therefore, longer T1 relaxation times represent more interstitial fibrosis. The association of T1 values has been histologically confirmed to predict myocardial fibrosis [28]. In chronically infarcted cardiac tissue, this represents interstitial fibrosis, which is also the common endpoint for RIHD [21, 24, 29–32].

In the current study, a CMR protocol including late gadolinium enhancement (LGE) and T1 mapping was used to evaluate local and diffuse fibrotic changes within the myocardia of patients who had previously received thoracic radiotherapy. Our hypothesis was that high dose regions of radiotherapy correlate to areas of LGE and T1 mapping on CMR. The purpose of this cross-sectional study was to determine the usefulness of CMR in the early detection of myocardial RIHD, with the eventual goal to develop methods for early intervention in the disease process.

## Materials and methods

### Patient selection

Any patient who had previously undergone radiotherapy for tumors within the thorax including lung, breast, esophagus cancer, and lymphoma was eligible after informed consent for enrollment in a cross-sectional protocol approved by our institutional review board, as long as the volume of heart receiving 5 Gy or more was at least 10% (V5Gy ≥ 10%). Patients were excluded if they had documented previous cardiac disease history, either prior to or after radiotherapy. In addition to CMR, patients received comprehensive cardiac evaluation, including cardiopulmonary exercise testing, baseline transthoracic echocardiograms, and laboratory evaluation, with results published previously [33].

### CMR acquisition

CMR was performed on 28 patients using the same 1.5 Tesla MRI scanner (Siemens Aera, Erlangen, Germany) and imaging protocol for all patients. ECG-gated imaging was performed as follows: balanced steady-state free precession (bSSFP) short axis cine images were obtained (6 mm thickness at 1 cm intervals, matrix of 256 X 200) from above the mitral valve through the left ventricular (LV) apex. Two-, 3-, and 4-chamber cine views were also obtained. T1 maps were obtained in three locations covering the left ventricle (base, mid-ventricle, and apex) using a Modified Look-Locker Inversion recovery (MOLLI) pulse sequence prior to contrast administration. T2 mapping images were obtained at similar locations. Contrast was administered using 0.2 mmol/kg of IV Prohance (Bracco Diagnostics Inc., Monroe Township, NJ). After a 2-min delay, long inversion time single-shot bSSFP inversion recovery images were obtained using a 550–600 ms inversion time. After 8–10 min, post-contrast images were obtained using an inversion time chosen to null normal myocardium. High-resolution gradient echo inversion recovery LGE images were obtained starting at 10 min post-contrast. These images were spatially matched to the cine images and covered the left ventricle. Post-contrast T1 mapping images were obtained at the end of the study. Post-processing was then performed using dedicated Precession imaging analysis software (Heart Imaging Technologies, Durham, NC).

### Image registration and data analysis

The analysis of LGE images was performed using Precession software and MIM image registration software (MIM Software Inc., Cleveland, OH). Computed tomography (CT) radiation therapy planning scans and corresponding radiotherapy plans were imported from radiation treatment planning software (Pinnacle, Phillips, Andover, MA) into MIM, in addition to the acquired CMR images. Cardiac structures including right and left ventricles, right and left atria, pericardium, and cardiac vasculature were contoured on CT by one radiation oncologist for consistency using a contouring atlas [34].

**Fig. 1:**
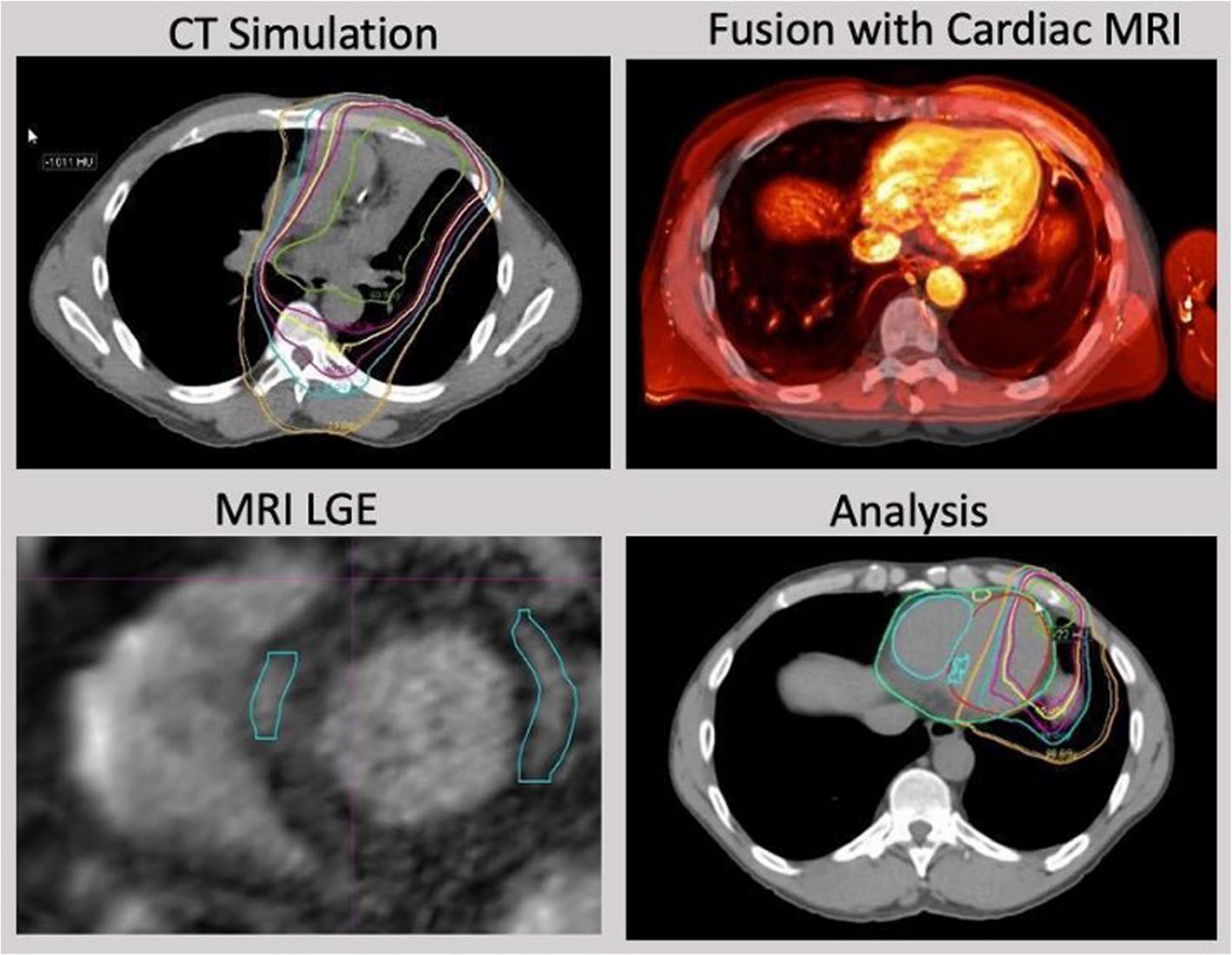
Image and dose registration process. Planning CT and associated radiotherapy treatment plan (top left) is rigidly registered with CMR (top right). Areas of LGE are delineated on CMR (bottom left). After image fusion, isodose distribution (colored lines represent areas of same dose) and LGE volume (blue volume within left ventricular septum, see arrow) are overlaid for dosimetric analysis

Three-dimensional rigid registrations of the left ventricles were performed fusing CMR to the CT simulation scans in MIM. A combination of auto-matching the left ventricle followed by manual adjustments was done to finalize the registration. An example of the rigid registration process is shown in Fig. 1. All rigid registrations were performed by the same dosimetrist for all patients on protocol.

The location and volume of LGE was evaluated within the left ventricular myocardium and septum. LGE volumes were well demarcated and easily visualized due to their higher signal compared to surrounding normal myocardium. For this study, the volume of LGE was delineated and LGE volumes were calculated by one radiation oncologist in conjunction with board certified radiologists who were blinded to the radiotherapy dose distribution. T1 values were calculated using Precession software by determining average T1 values (relaxation time measured in ms) using the mid-ventricle short axis slice which showed larger ventricle thickness and had higher spatial reproducibility compared to the apex and base slices.

### Dose analysis

All patients had T1 mapping and LGE sequences acquired. Dosimetric analysis was undertaken to explore if a dose response relationship existed between cardiac dose and both left ventricular T1 values and areas of LGE. Visualization of isodose distributions and areas of LGE were performed on the axial CT plane (Fig. 1) after superimposing the volume of LGE on the CT planning scan. If patients received more than one radiotherapy course, composite radiotherapy plans were created in MIM that incorporated the total number of courses. Dose-volume histograms were calculated for LGE volumes in addition to the whole heart and left ventricle. Specific variables included the volume of enhancement (LGE volume), mean and maximum dose to the enhancing volume (LGE Dmean and LGE Dmax), mean and maximum dose to the heart (heart Dmean and heart Dmax), mean and maximum dose to the left ventricle (LV Dmean and LV Dmax), minimum dose to 95% of the LGE volume (D95), and the volume of heart and volume of left ventricle receiving defined radiation doses (V5/30/40/50Gy). Due to varying fractionation schedules between patients and for repeated courses in the patients indicated in Table 1, all doses were converted into equivalent doses to 2 Gy per fraction schemes (EQD2) using an alpha/beta ratio of 2 Gy for heart [20, 35].

**Table 1:** Patient characteristics. Numbers are expressed as mean values (range) or number (%) as appropriate

Characteristic	Mean values, unless specified differently (range)
Age (years)	62 (28–87)
Primary site (#)	14 (50%) lung
	9 (32%) breast
	2 (7%) esophagus
	1 (4%) lymphoma
	2 (7%) other
Gender (#)	14 (50%) male
	14 (50%) female
Time to CMR (months)	46.4 (1.7–344.5)
Smoking History (#)	16 (57%) yes
	12 (43%) no
Hypertension (#)	16 (57%) yes
	12 (43%) no
Body Mass Index (kg/m^2^)	27.7 (16.0–50.6)
Radiotherapy technique (#)	15 (54%) 3D conformal
	11 (39%) IMRT
	2 (7%) SBRT
Total radiotherapy courses (#)	23 (82%) one course
	4 (14%) two courses
	1 (4%) three courses
Chemotherapy (#)	13 (43%) concurrent
	9 (32%) neoadjuvant/adjuvant
	7 (25%) none
Adriamycin use (#)	7 (25%) yes
	21 (75%) no

*CMR* cardiac magnetic resonance imaging, *3D* 3D conformal therapy, *EQD2* equivalent dose in 2Gy fractions, *IMRT* intensity modulated radiation therapy, *SBRT* stereotactic body radiation therapy, *V5Gy* percentage volume of heart receiving at least 5Gy, # number of patients

### Statistical analysis

At the initiation of our study, no reports were available on the radiation dose dependence of LGE. Therefore, no guidance was available to determine the cohort size and statistical power. This study was designed primarily to be a hypothesis-generating observational study.

Nonetheless, several associations were analyzed as follows: We performed univariate comparisons tests (i.e., t-tests and Fisher exact tests for continuous and discrete variables, respectively), with attention to heart dosimetric differences (discussed above) and clinical factors among patients both who did and did not experience LGE. We specifically considered differences in several oncology-relevant factors, including tumor site (lung, breast, or other), body mass index, smoking status, hypertension diagnosis, and Adriamycin use. Finally, we used a two-sided t-test to consider the association between T1 mapping values and LGE. We also performed a linear regression using the ordinary least squares method to correlate Dmax and Dmean doses to T1 values. Microsoft Office Excel 2016 (Redwond, WA) and Stata MP 15 (StataCorp LLC, College Station, TX) were used for statistical analyses.

## Results

The cohort included 28 patients of which the majority had lung and breast cancer (see Table 1). The mean time from end of radiation treatment to CMR acquisition was 46.4 months (range 1.7–344.5). All patients had a heart V5Gy volume of at least 10%. The majority of patients received chemotherapy (75%) either neoadjuvantly/adjuvantly (9 patients) or concurrently (12 patients). Chemotherapy drugs included adriamycin in 7 patients and trastuzumab in one patient, with most patients receiving carboplatin and paclitaxel as part of lung cancer treatments. Six patients received more than one radiation treatment course and cumulative dose composite plans were created.

The Dmax and Dmean EQD2 doses to the heart for the entire cohort were on average 50.9 Gy (range 6.2–108.0) and 8.2 Gy (range 1.0–35.7), respectively. As the T1 value analysis was focused on the left ventricle and septum, Dmax and Dmean EQD2 doses to the left ventricle were analyzed as well. These doses were on average 34.0 Gy (0.2–94.7) and 8.2 Gy (0.1–34.4), respectively. Further patient characteristics are presented in Table 1, additional dose characteristics are shown in Table 3.

### Late gadolinium enhancement

Nine patients demonstrated areas of LGE, all within their left myocardium or septum. These 9 patients provided the data discussed below, with their treatment characteristics listed in Table 2. Patients demonstrating enhancement had a median interval between radiotherapy and CMR of 11.9 months compared to 31.4 months in patients without enhancement (*p* = 0.33).

**Table 2:** Characteristics of patients demonstrating LGE

Patient number	Age	Time from EOT to CMR (mos)	Cancer site	LGE volume (cc)	Dmean EQD2 (Gy) to LGE	Dmean EQD2 (Gy) to heart	Dmax EQD2 (Gy) to LGE	Dmax EQD2 (Gy) to heart	D95 EQD2 (Gy) to LGE
1	87	28.3	lung	2.4	6.7	4.5	28.2	41.3	0.7
2	50	7.7	lung	0.2	16.5	4.7	25.5	48.8	0.9
3	67	22.1	lung	5.5	12.0	6.8	43.1	108	1.3
4	67	14.2	breast	1.0	12.4	1.8	16.1	40.8	8.7
5	65	2.2	breast	6.1	1.7	2.3	3.7	51.3	0.9
6	60	11.9	lung	2.2	0.4	4.1	0.5	66.6	0.3
7	71	26.5	esophagus	2.1	6.1	8.8	16.6	51.9	2.2
8	56	2.2	lung	0.5	10.9	21.8	13.2	69.9	9.1
9	74	6.0	lung	0.7	0.6	6.2	0.7	68.8	0.5
Average (Range)	66 (50–87)	13.4 (2.2–28.3)	–	2.3 (0.2–6.1)	7.5 (0.4–16.5)	6.8 (1.8–21.8)	16.4 (0.5–43.1)	60.8 (40.8–108.0)	2.7 (0.3–9.1)

* *Cc* cubic centimeters, *CMR* cardiac magnetic resonance imaging, *Dmax* maximum dose, *Dmean* mean dose, *D95* minimum dose to 95% of the LGE volume, *EOT* end of treatment date, *EQD2* equivalent dose in 2Gy fractions, *Gy* Gray, *LGE* late gadolinium enhancement, *mos* months

Among patients who developed LGE, the mean volume of LGE was 2.3 ml (0.2–6.1). The cardiac EQD2 Dmax and Dmean in patients with LGE were on average 60.8 Gy (40.8–108.0) and 6.8 Gy (1.8–21.8), respectively. The EQD2 Dmax and Dmean to the left ventricle were on average 43.8 Gy (13.6–94.7) and 7.6 Gy (1.4–34.4), respectively. The 19 patients without LGE had cardiac EQD2 Dmax and Dmean on average of 46.1 Gy (6.2–82.6) and 8.8 Gy (1.0–35.7), respectively. Among these patients, the cardiac EQD2 Dmax and Dmean to the left ventricle were on average 29.4 Gy (0.2–74.7) and 8.4 Gy (0.1–30.0), respectively. Between individuals with and without LGE, no significant difference was seen in EQD2 Dmax or Dmean total heart doses (*p* = 0.16 and 0.57, respectively) and EQD2 Dmax or Dmean of the left ventricle (*p* = 0.17 and 0.84, respectively). Further dosimetric parameters including heart and left ventricle V5Gy/V30Gy/V40Gy/V50Gy, as well as D95 to the LGE volume, were also investigated. No significant difference was identified between patients with and without LGE (see Table 3).

**Table 3:** Dosimetric data evaluating LGE

Factor (mean (range))	All patients (*N* = 28)	No LGE (*N* = 19)	LGE (*N* = 9)	*p*-value
Dmax Heart EQD2 (Gy)	50.9 (6.2–108.0)	46.1 (6.2–82.6)	60.8 (40.8–108.0)	0.16
Dmean Heart EQD2 (Gy)	8.2 (1.0–35.7)	8.8 (1.0–35.7)	6.8 (1.8–21.8)	0.57
Dmax LV EQD2 (Gy)	34.0 (0.2–94.7)	29.4 (0.2–74.7)	43.8 (13.6–94.7)	0.17
Dmean LV EQD2 (Gy)	8.2 (0.1–34.4)	8.4 (0.1–30.0)	7.6 (1.4–34.4)	0.84
Heart V5Gy (%)	48.5 (10–97)	49.3 (10–97)	46.8 (13–96)	0.85
Heart V30Gy (%)	10.8 (0–69)	11.0 (0–66)	10.5 (0–44)	0.94
Heart V40Gy (%)	7.6 (0–66)	8.1 (0–66)	6.7 (0–33)	0.83
Heart V50Gy (%)	5.1 (0–59)	5.8 (0–59.0)	3.4 (0–23)	0.65
LV V5Gy (%)	40.1 (0–100)	37.3 (0–100)	45.1 (18.5–100)	0.61
LV V30Gy (%)	10.7 (0–71.8)	10.7 (0–65.1)	10.7 (0–71.8)	0.99
LV V40Gy (%)	7.7 (0–55.5)	7.8 (0–55.5)	7.4 (0–53.1)	0.95
LV V50Gy (%)	5.1 (0–48.8)	5.6 (0–48.8)	4.3 (0–36.4)	0.81
T1 mapping values (ms)	1009 (933–1117)	997 (933–1067)	1033 (967–1117)	0.054

*Dmax maximum dose, *Dmean* mean dose, *EQD2* equivalent dose in 2 Gy fractions, *Gy* Gray, *LGE* late gadolinium enhancement, *LV* left ventricle, *ms* milliseconds, *Vx* volume in % receiving ≥ dose x in Gy. *P*-values reported for significance testing among patients with and without LGE

The EQD2 Dmax and Dmean to the LGE volume itself was 16.4 Gy (0.5–43.1) and 7.5 Gy (0.4–16.5), respectively, which were lower or similar compared to whole heart and left ventricle doses. D95 doses of the LGE volumes also clearly show that doses in these volumes were in general low. The location of the maximum heart dose was located outside the contoured LGE volume in all 9 patients. There was no geographic relationship between dose to coronary vessels and downstream myocardial areas of enhancement. There was no association between EQD2 Dmax and Dmean in the LGE area and the size of the LGE volume (*p* = 0.13 and *p* = 0.78, respectively). Patients who had evidence of LGE also had a trend towards higher T1 values (*p* = 0.054, Table 2).

### T1 mapping

**Fig. 2:**
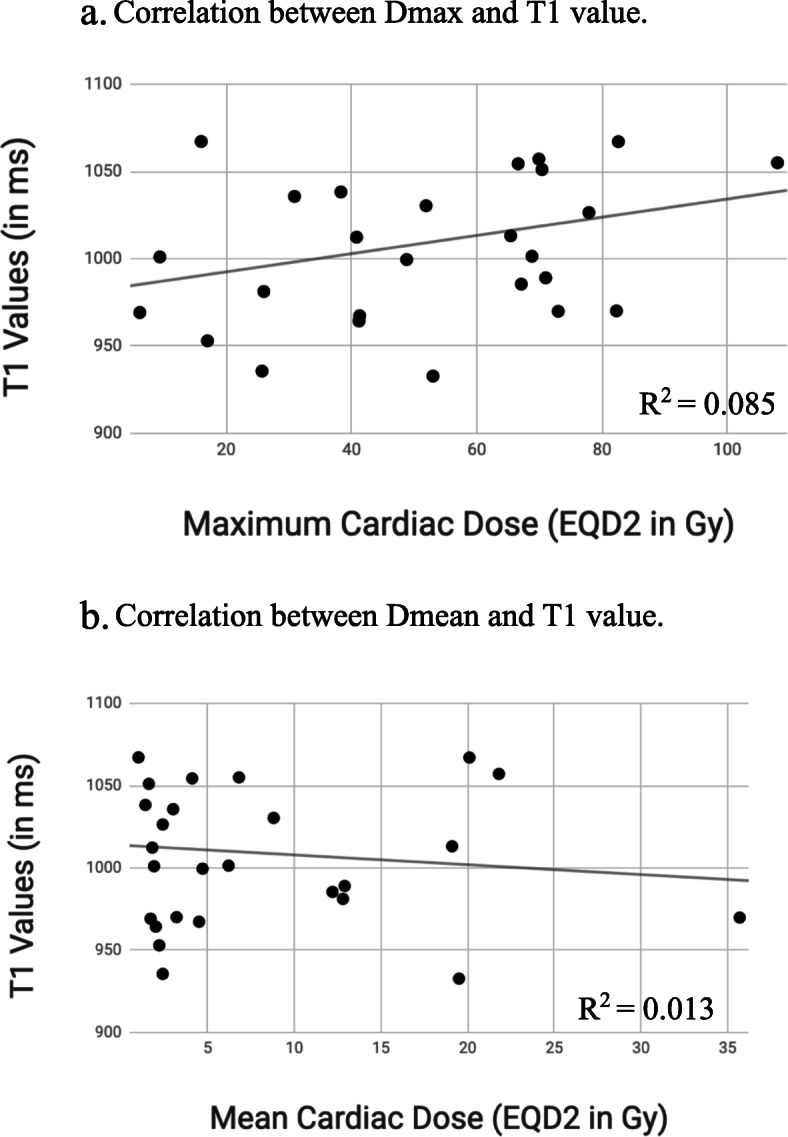
Correlations between cardiac Dmax and Dmean and T1 values. **a**. Correlation between Dmax and T1 value. **b**. Correlation between Dmean and T1 value. No significant correlation between heart dose and T1 values is seen (*p* > 0.05)

All patients had diffuse T1 mapping performed. The average T1 value of the left myocardium and septum of patients on study was 1009 ms (range 933–1117). The association between T1 values and the heart and left ventricle EQD2 Dmax and Dmean values were analyzed. Linear correlations between heart Dmax and Dmean (in EQD2 Gy) and T1 mapping values (in ms) were also evaluated (Fig. 2). No significant correlation was seen for heart or left ventricle Dmax or Dmean and T1 values in bivariate models (heart *p* = 0.139 and 0.575, LV *p* = 0.393 and 0.613 respectively).

## Discussion

There is a great need to identify patients at risk of RIHD after radiotherapy in order to institute preventive measures and early interventions. It was with the goal of determining the usefulness of CMRs in the detection of preclinical RIHD that the current pilot study was undertaken. CMR appeared to be a promising candidate towards a non-invasive screening test in patients at high risk for RIHD[21–26, 28]. Despite long intervals and high radiation doses in several patients in our study, no association between radiation dose and myocardial changes was identified using these techniques.

The current study did not demonstrate significant correlations between LGE and radiation doses. We did not observe a dose-volume relationship between previously delivered dose distributions and areas of LGE. Included in our analysis was a wide range of known predictive factors for RIHD, including whole heart and left ventricle V5Gy, V30Gy, V40Gy, and V50Gy according to data from Bradley et al., Chun et al., Bogaard et al., and Speirs et al. [13, 15, 36, 37] All were found to not be significantly correlated with LGE presence. The location of cardiac Dmax was located outside the LGE volume in all patients with demonstrated LGE. There was also no association between cardiac Dmax and Dmean and the LGE volume. Based on this study, myocardial fibrosis and tissue remodeling following radiation treatment may not occur in high dose regions of radiation treatment plans.

In addition, diffuse T1 mapping did not show a clear dose-response relationship despite high cardiac radiation doses and multi-year follow up in some patients. There was a trend towards higher T1 values in patients with evidence of LGE, which likely shows the effect of local fibrosis changes on mean T1 values obtained from larger volumes.

On our review, the current study is one of only few to evaluate LGE and T1 mapping sequences in the detection of diffuse left ventricular fibrosis secondary to radiotherapy. Our analysis on LGE most similarly parallels the recently published manuscript by Huang et al. [38] In Huang et al., 7 patients with previous thoracic radiotherapy with median time from treatment to CMR of 3.1 years exhibited a linear relationship between EQD2 Dmean and Dmax doses delivered to the left atrium and right atrium and the fibrosis volume on CMR. Interestingly, however, they did not observe any focal left or right ventricle myocardial fibrosis, and did not see a dose relationship within the right atrium scar volume [38]. In our LGE protocol, no images of the right or left atria were acquired as atrial walls are generally thin and reliable LGE measurement is therefore difficult. Despite higher prescription doses in our study no dose effect was identified for LGE. This is likely due to all 7 patients on the Huang study having received simple mediastinal 3D treatments with homogeneous dose delivered to large parts of the left and right atrium, compared to the current study where the majority of patients received heterogeneous, often much higher doses than in the Huang study delivered to small volumes of the heart. These findings suggest that the volume of irradiated myocardium may play a role in development of fibrosis. In addition, there is evidence of differing radiosensitivity of different cardiac substructures, namely the bilateral atria and great vessels, potentially affecting LGE distribution [39].

In a study by Umezawa et al. [40], 24 esophageal cancer patients who were treated with concurrent radio-chemotherapy to 66–70 Gy received CMRs at a median time of 23.5 months after completion of radiotherapy. All patients had 3D conformal treatments. Fifty percent of those patients demonstrated LGE, with LGE being localized always within the segments within either the predominantly 40Gy (15.38% with positive LGE of all 40Gy segments) or 60Gy (21.21% with positive LGE of all 60Gy segments) isodose distributions. In a follow up study by the same group, Takagi et al. prospectively enrolled 14 patients with newly diagnosed esophageal cancer, with serial CMRs taken before, 0.5 years and 1.5 years after 50.4–57.4Gy chemoradiotherapy treated with 3D conformal techniques. LGE was detected in one baseline pretreatment CMR, with 78% (11/14) demonstrating LGE 1.5 years after chemoradiotherapy, mostly detected in the basal septum corresponding to high dose regions. While cardiac radiation doses were not stated, given 3D conformal treatments and esophageal cancer primaries, patients on the Umezawa et al. and Takagi et al. studies likely had higher mean cardiac doses than our cohort, as well as larger and more homogeneously irradiated cardiac volumes, similar in characteristics to patients on the above Huang et al. study.

Even less information is available on T1 mapping and RIHD. A case study describes a 70 year old male treated with chemoradiotherapy for esophageal cancer, who developed LV systolic failure 8 years after cancer treatment. CMR detected a T1 time of 1303 ms, which was the upper limit of reference range per the authors’ institution. Confirmatory endocardial biopsy showed interstitial fibrosis and myocardial degeneration compatible with RIHD [41]. The significance of this T1 value is unclear, as there is a lack of standardization and a true reference range is currently not established. Individual T1 values can not be compared between studies due to differences between vendors, CMR sequences, and post-processing [26, 42]. Tuohenin et al. studied 20 patients with early stage left-sided breast cancer received CMR with T1 mapping 6 years after radiotherapy. Diffuse T1 relaxation times were on average 1210 ms (+/− 52 ms) within inferoseptal segments of left ventricles which corresponded to radiation treatment fields, 35% of T1 values in this region were greater than 1250 ms, significantly greater than in other regions [43].

The most comprehensive study on T1 mapping in the detection of RIHD is also by Takagi et al. as described above. Mean T1 values for CMR at baseline prior to radiotherapy were 1183 ms (+/− 46); however, values taken at the basal septum which received sizeable radiation doses were significantly elevated above baseline pretreatment values (0.5 years = 1257 ms (*p* < 0.01), 1.5 years = 1238 ms (*p* = 0.024)). Interestingly, the left lateral ventricle segment, which would have been outside of the traditional 3D conformal field and received only low dose, did not see differences between pre-treatment and post-treatment T1 values [44].

The time to the development of cardiac fibrosis is not known, but it is assumed to develop within 6 months after radiotherapy and is not reversible once deposited. The median time from end of treatment to CMR acquisition in our sample was 24.6 months, similar to the studies by Huang et al. and Umezawa et al. who detected fibrosis at a median 37.2 months and 23.5 months following radiotherapy, respectively, which confirms this assumption. Patients demonstrating enhancement had a median interval between radiotherapy and CMR of 11.9 months compared to 31.4 months in patients without enhancement (*p* = 0.33), but the significance of this absolute difference is unclear and likely subject to bias and confounders.

There are several limitations to the current study. While RIHD can have many causes, CMR in this study primarily assessed myocardial damage and therefore cardiomyopathy. Due to the cross-sectional study design, no pre-radiotherapy CMRs were acquired and therefore areas of LGE on post-treatment CMRs may actually be related to “silent” undiagnosed cardiac events or may have been pre-existing. In addition, we cannot exclude that chemotherapy might have been related to LGE development. Although not demonstrated in the present study, with radiotherapy being a localized treatment a correlation between the location and volume of the LGE with the high dose distribution was anticipated whereas for systemic treatments more diffuse myocardial effects would be expected. While ideally investigating one primary tumor site only would have provided a more homogeneous patient cohort, dose standardization and investigation of a large spectrum of dose parameters in the present study are expected to account for interpatient differences in dose distribution and treatment techniques. Image fusion in this study was completed with rigid registrations; given the time interval between CT simulation and CMR, patient anatomy might have changed. To account for these anatomical changes, rigid registrations included rotations, translations and changes in magnitude aligned to the organ of interest, namely the left ventricle. The power of the current study is limited due to its small sample size. With the large variations in cardiac dose in our study, any clinically relevant relation between cardiac dose and myocardial fibrosis or LGE development should have been noticeable despite the limited sample size. In fact, patients with LGE in our study typically had lower maximum and mean doses compared to no-LGE patients. In addition, for all investigated dose levels, heart and left ventricle volumes were in general smaller for patients with LGE than without LGE development. Evaluation of LGE and T1 values on longitudinal studies are expected to further clarify the effect of radiotherapy on myocardial changes.

## Conclusion

In this pilot study, no relationship between cardiac radiation dose and localized fibrosis was seen using LGE on CMR. There was also no association between cardiac doses and T1 values as a measure of diffuse fibrosis. Further study, including volumetric CMR imaging and longitudinal assessment, is needed to determine the benefit of cardiac MRI in the detection of myocardial fibrosis following radiotherapy.

## Data Availability

The datasets during and/or analyzed during the current study available from the corresponding author on reasonable request.
